# Early depressive symptoms and disability accrual in Multiple Sclerosis: a UK MS Register study

**DOI:** 10.1038/s41598-023-34545-6

**Published:** 2023-05-24

**Authors:** Benjamin M. Jacobs, Cyrus Daruwalla, Mollie O. McKeon, Raghda Al-Najjar, Andrea Simcock-Davies, Katherine Tuite-Dalton, J. William L. Brown, Ruth Dobson, Jeff Rodgers, Rod Middleton

**Affiliations:** 1 Preventive Neurology Unit, Wolfson Institute of Population Health, Queen Mary University of London, London, UK; 2 Department of Neurology, Royal London Hospital, London, UK; 3 Department of Clinical Neurosciences, University of Cambridge, Cambridge, UK; 4 Population Data Science, Swansea University Medical School, Swansea, UK

**Keywords:** Multiple sclerosis, Epidemiology

## Abstract

Understanding the associations and potential drivers of long-term disability in Multiple Sclerosis (MS) is of clinical and prognostic value. Previous data have suggested a link between depression and disability accrual in MS. We aimed to determine whether depression in early MS predicts subsequent accrual of disability. Using data from the UK MS Register, we identified individuals with and without symptoms of depression and anxiety close to disease onset. We used Cox proportional hazards regression to evaluate whether early depressive or anxiety symptoms predict subsequent physical disability worsening, measured using the Expanded Disability Status Scale (EDSS). We analysed data from 862 people with MS of whom 134 (15.5%) reached an EDSS of ≥ 6.0. Early depressive symptoms were associated with an increased risk of reaching an EDSS of 6.0 (HR 2.42, 95% CI 1.49–3.95, p < 0.001), however this effect dissipated when adjusting for baseline EDSS (HR 1.40, 95% CI 0.84–2.32, p = 0.2). These data suggest that early depressive symptoms in MS are associated with subsequent disability accrual, but are likely the result of disability rather than its cause.

## Introduction

Multiple Sclerosis (MS) is a common cause of neurological disability among young adults^1^. The natural history of MS varies between individuals, but the determinants of this heterogeneity are incompletely understood. Factors that appear to favour a more benign MS course include later age at onset, relapse-onset disease, female sex, not smoking, early use of high-efficacy therapies, genetic factors, and lack of vascular comorbidities^2–6^. Understanding the drivers of long-term disability in MS may suggest novel therapeutic strategies for slowing the progression of the disease.

Psychiatric comorbidities such as anxiety disorders and depression are common in people with MS^7,8^, with up to 50% experiencing depression at some point. There have been few longitudinal studies examining the association between depression and subsequent disability outcomes in MS. A Dutch study found no association between depression at baseline and subsequent disability worsening^9^, whereas larger and more recent studies conducted in Canada^10^ and Sweden^11^ found that individuals with MS and depression were more likely to reach disability milestones than those without depression. Clarifying the nature of this association has implications for the management of comorbid depressive symptoms in MS. We aimed to explore the association between psychiatric comorbidity—depression and anxiety—and subsequent disability accrual in a longitudinal UK cohort, the UK MS Register (UKMSR).

## Methods

### Cohort definition

The UK MS Register (UKMSR) is a longitudinal cohort study of people with Multiple Sclerosis living in the UK^12^. Since 2011, the UKMSR has collected online self-reported information on demographics, risk factors, MS-related outcomes, and validated patient-reported outcomes via an online portal. For a subset of participants enrolled at clinical sites, a clinical minimum dataset is captured. In this study we used the patient-reported outcome (PRO) data as the primary data source. Data were accessed in June 2022.

### Variable definitions

Expanded Disability Status Scale (EDSS^13^) values were extracted from two sources: a validated online self-administered EDSS scale (the webEDSS^14^), and clinician-determined EDSS available for a subset of the population. The primary outcome was defined as the probability of reaching EDSS ≥ 6.0. EDSS 6.0 reflects use of a unilateral walking aid, and is frequently used as an endpoint in observational studies. All other data used in this study were solely derived from participant-reported measures.

Anxiety and depression were defined using the Hospital Anxiety and Depression Scale (HADS)^15^, which UKMSR participants are encouraged to complete online via the web portal at 6 monthly intervals from the time of registration. HADS is one of a battery of PRO measures regularly ascertained via the UKMSR. Anxiety and depression were dichotomised using the established cutoff of 7^15^. We classified individuals as ‘anxious’ or ‘depressed’ if one of these respective scores was > 7, i.e. 8 or higher. Only HADS scores obtained before the baseline EDSS recording or in the 6 months following the baseline EDSS recording were considered, and the HADS score closest to their baseline EDSS recording was used.

### Inclusion and exclusion criteria

We defined the study population using the following inclusion criteria, all of which had to be satisfied for inclusion in the analysis:

- Complete demographic data (year of birth, year of diagnosis, gender, MS type at diagnosis);
- At least one ‘baseline’ EDSS value within 5 years of self-reported MS diagnosis date;
- At least one subsequent EDSS recording more than 6 months after the baseline reading;
- Baseline EDSS score < 6.0;
- At least one HADS score before or within 6 months of the baseline EDSS reading.

**Figure 1:**
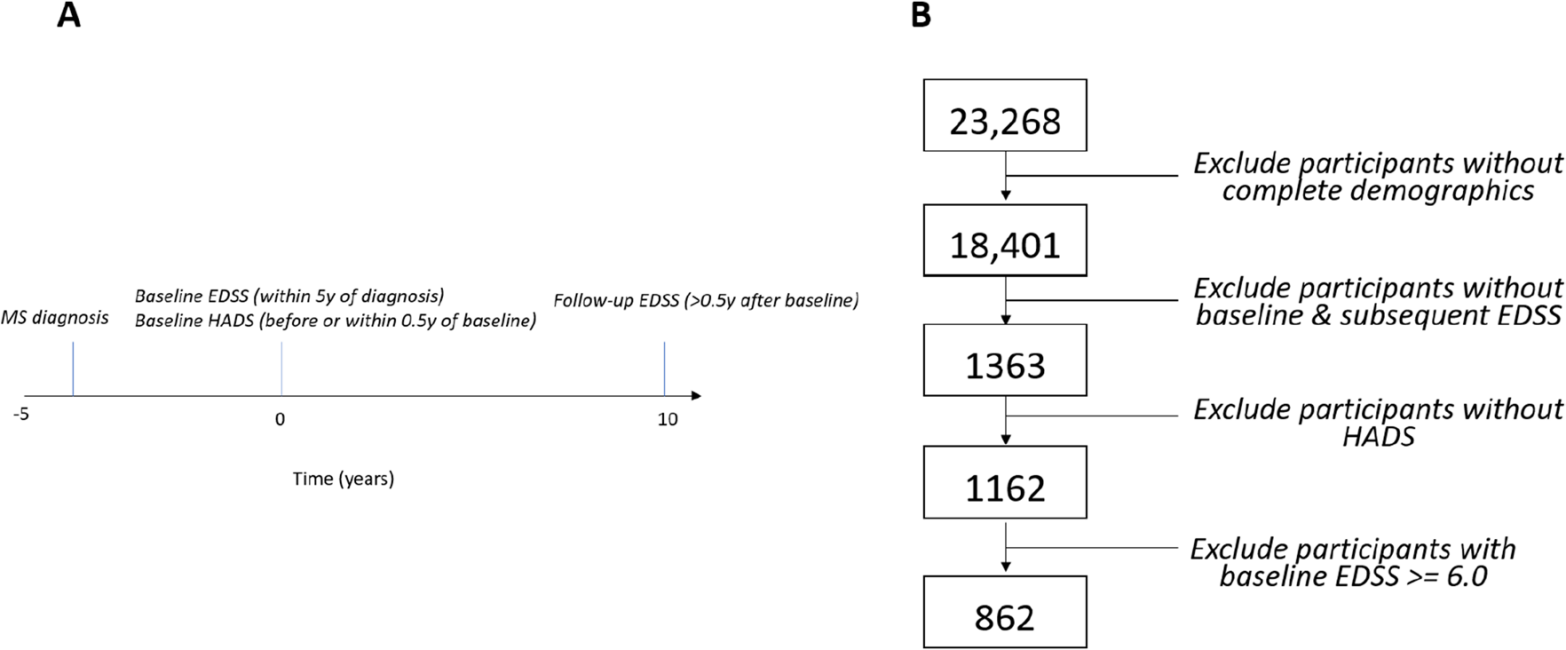
Study design. (**A**) study design. Baseline EDSS scores were ascertained within 5 years of MS diagnosis. Baseline HADS scores were ascertained before or within 6 months of the baseline EDSS. Follow-up EDSS scores recorded at least 6 months after the baseline EDSS were considered. Within the follow-up period we classified participants according to whether they reached EDSS 6.0 or not. (**B**) PRISMA flow diagram showing the number of participants included in the study population after application of inclusion and exclusion criteria.

From the entire UKMSR population (n = 23,268), we defined the analysis population for the study as those individuals with complete baseline demographic data (n = 18,401), at least one ‘baseline’ EDSS reading (i.e. an EDSS reading within 5 years of their stated MS diagnosis date), one or more EDSS readings at least 6 months after their baseline reading, a valid HADS score before or in the 6 months after their baseline EDSS reading, and a baseline EDSS reading of < 6.0 (Fig. 1). Figure 1 depicts the number of individuals excluded at each stage of filtering (Fig. 1). Baseline EDSS were ascertained at a median of 2.4 years after MS diagnosis and 5.3 years after symptom onset.

### Statistical analysis

We used Spearman rank tests and chi-squared tests for univariable comparisons of variables between individuals who reached EDSS 6.0 and those who did not. Unless specified, categorical variables are reported as n (%) and continuous variables as median (interquartile range). To determine the association between anxiety and depression at baseline and time to EDSS 6.0, we used Cox proportional hazards models. In the primary analysis, we adjusted for age at baseline EDSS, gender, and Primary Progressive MS (PPMS) vs other MS types. We conducted a range of secondary sensitivity analyses using the following model specifications:

- Primary Analysis: age at baseline EDSS + Gender + PPMS-onset
- Secondary analyses﻿:
- As for primary analysis + disease duration at time of EDSS
- As for primary analysis + baseline EDSS
- Just Relapsing–Remitting MS (RRMS), adjusted for age at baseline EDSS + Gender
- Just males, adjusted for age at baseline EDSS + PPMS-onset
- Just females, adjusted for age at baseline EDSS + PPMS-onset
- Just participants with baseline EDSS < 4.0, covariates as per primary analysis
- Age at baseline EDSS + Gender + exposure to high-efficacy disease modifying therapy (DMT) (alemtuzumab, cladribine, ocrelizumab, fingolimod, and natalizumab) prior to baseline EDSS recording. Individuals with no DMT data were considered to be unexposed to high-efficacy DMT.

We used Schoenfeld residuals to check the validity of the proportional hazards assumption, checked for unduly influential observations, and checked the linearity of the relationship between continuous covariates and disability progression.

All analyses were conducted in R version 4.1.3 within the UKMSR secure research environment (UKSERP^16^).

### Ethical approval

The UK Multiple Sclerosis Register has research ethics approval from South West Central Bristol Research Ethics Committee (initially as 16/SW/0194, currently 21/SW/0085). This project was approved as part of the UKMSR Datathon. All methods were performed in accordance with the relevant guidelines and regulations. Informed consent was obtained from all participants prior to participation.

## Results

We analysed data from 862 persons with MS from the UK MS Register (76.3% female, median age at diagnosis 43.0 [IQR 17.0], median age at symptom onset 38.0 [IQR 17.0], 83.1% relapsing–remitting MS (RRMS), Fig. 1). Of this cohort, 134 people (15.5%) reached an EDSS of 6.0 or more during the follow-up period (Table 1). The median age at MS diagnosis was higher in those who reached EDSS 6.0 (median 48.5 vs median 42.0, p < 0.001; Table 1). The proportion of individuals with a diagnosis of Primary Progressive MS (PPMS) at diagnosis was higher among those who progressed to EDSS 6.0 (23.0% vs 7.6%, p < 0.001), as was the baseline EDSS (median 4.5 vs median 3.0, p < 0.001). Those who reached EDSS 6.0 also tended to be older at the time of their baseline EDSS recording (median 50.9 years vs median 45.1 years, p < 0.001; Table 1). Linked relapse data from clinical records were available for 84 participants, of whom 19/84 (22.6%) had a recorded relapse within 1 month of their baseline HADS reading.

**Table 1:** Demographic characteristics and key variables of the study population are shown.

Variable	Did not reach EDSS 6.0	Reached EDSS 6.0	P value
N	728	134	
Age at diagnosis	42.0 (34.0–50.0)	48.5 (41.0–54.0)	< 0.001
Age at symptom onset	37.0 (30.0–46.0)	42.0 (33.0–50.0)	0.002
Age at baseline EDSS	45.1 (35.8–52.6)	50.9 (43.2–56.1)	< 0.001
Gender (female)	561 (77.1%)	98 (73.1%)	0.382
PPMS	53 (7.6%)	29 (23.0%)	< 0.001
Baseline EDSS	3.0 (2.0–4.0)	4.5 (3.6–5.0)	< 0.001
Exposed to high-efficacy DMT prior to baseline EDSS	112 (15.4%)	9 (6.7%)	0.012
Depression at baseline	163 (22.4%)	58 (43.3%)	< 0.001
Anxiety at baseline	298 (40.9%)	62 (46.3%)	0.29

Demographics are shown for individuals who reached EDSS 6.0 during the study follow-up period and those who did not. P values reflect univariable statistical comparisons between the two groups.

We examined the differences in demographic characteristics between our study population and the wider UKMSR population (n = 17,539) to assess the risk of collider bias. The study population was diagnosed at an older age (median 43.0 vs 38.0, p < 0.001), with a roughly similar gender split (76.5% female vs 74.5%, p = 0.2), a higher proportion of people with RRMS compared to the non-study cohort (83.1% vs 72.5%, p < 0.001), and were diagnosed more recently (median 2016 vs 2007, p < 0.001).

Anxiety and depression were common in both groups. The proportion of people with anxiety at baseline did not differ significantly between those who reached EDSS 6.0 and those who did not (46.3% vs 40.9%, p = 0.29), whereas depression at baseline was more common among those who reached EDSS 6.0 (43.3% vs 22.4%, p < 0.001).

**Figure 2:**
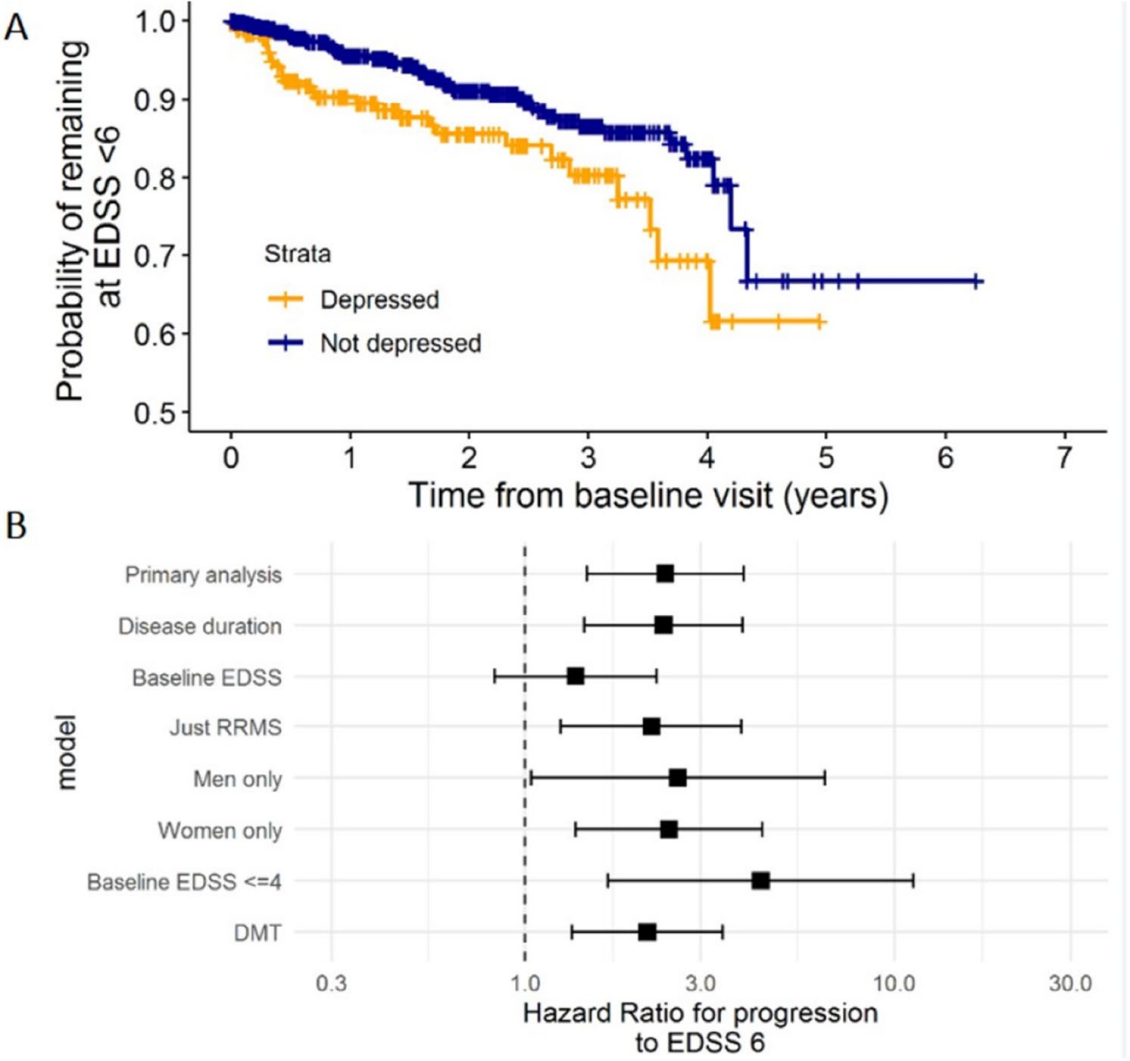
Association between depressive symptoms and disability accrual in Multiple Sclerosis. (**A**) Kaplan–Meier curves showing the probability of survival free from progression to EDSS 6.0 within the study observation period (> 5 and < 10 years after diagnosis) for individuals with and without depression (HADS depression score ≥ 8) at baseline. (**B**) Forest plot depicting Hazard Ratios (HRs) and 95% confidence intervals for the hazard of progression to EDSS 6.0 given the presence or absence of depression at baseline. The dashed line at 1 indicates the null. The x axis is on a log-10 scale. These HRs were derived from a variety of multivariable cox proportional hazards models. The y axis describes the model used. We performed a variety of sensitivity analyses including using different confounding covariates, outcome definitions, and cohort definitions. ‘Primary analysis’ refers to the primary analysis model, in which we adjusted for age at baseline EDSS recording, MS subtype (PPMS vs other types), and gender.

Depression at baseline was associated with an increased hazard of reaching EDSS 6.0 (Multivariable Cox regression models adjusted for age at EDSS measurement, gender, and MS subtype; HR 2.42, 95% CI 1.49–3.95, p < 0.001; Fig. 2A). We observed similar effects in sensitivity analyses, including adjustment for disease duration, sex-stratified analyses, analyses including only participants with relapse-onset MS, and adjustment for exposure to high-efficacy disease-modifying therapy. However, adjustment for baseline EDSS diminished the effect (HR 1.40, 95% CI 0.84–2.32, p = 0.20; Fig. 2B). We did not observe a statistically significant association between anxiety at baseline and subsequent EDSS 6.0 (HR 1.49, 95% CI 0.92–2.41, p = 0.10).

## Discussion

We provide evidence from the UK MS Register for a relationship between depression and subsequent disability accrual in MS, corroborating previous findings from Swedish^11^ and Canadian cohorts^17^. Importantly, this relationship dissipates almost entirely on adjustment for baseline disability.

There are several plausible explanations for these data: psychiatric illness could be triggered by physical disability, could stem from a common causal origin (i.e. a higher brain lesion load may predispose to depression/anxiety and physical disability), or could arise independently but contribute to overall disability via a separate process. Adjusting for baseline EDSS substantially diminished the signal, suggesting that depression may simply reflect more advanced disease rather than being an independent predictor of progression. An important limitation of this work is the relatively small proportion of the cohort with serial EDSS measures and HADS scores, which raises the possibility of collider bias as both increasing physical disability and depression are likely to influence an individual’s probability of participating in these assessments. Furthermore, the use of self-reported EDSS measures, although broadly accurate, may be less accurate than clinician-determined EDSS. Both HADS and EDSS scores fluctuate over time, for instance during a relapse (often termed relapse-associated worsening); while the small proportion of patients with a recorded relapse near the time of HADS recording is reassuring, relapse data were only available for a minority of the cohort, and therefore this is a weakness of the present study.

Given the stringent filtering we applied from the broader cohort, it is plausible that collider bias could distort the results, for instance potentially disguising a genuine association and leading to a false negative result. Our study has focussed on a more recently-diagnosed, younger, and more heavily relapse-onset cohort than the whole UKMSR population. By definition, individuals who are able to complete the webEDSS are likely to be skewed towards individuals with less aggressive disease, more preserved upper limb and cognitive function. Furthermore, depression itself is likely to influence both the completion of the HADS score and the webEDSS score, and so it is likely that collider biases have an impact on these findings.

As participants are enrolled after being diagnosed with MS, we did not have data on premorbid psychiatric symptoms. This precludes any comment on the temporal relationship between depressive symptoms and MS-related disability based on these data alone. For parsimony we considered the presence or absence of psychiatric comorbidity at baseline as a static predictor of future disability, however this approach does not account for fluctuations in psychiatric symptoms over time. We were unable to fully address the influence of disease-modifying therapy (DMT) or of relapses on disability accrual due to data availability. Although we do adjust for DMT exposure, these data are only available for a small subset of participants and so this analysis is unlikely to fully capture the effect of treatment.

In this study, we replicate the previously-observed association between depressive symptoms and subsequent disability progression. Our results raise the suspicion that depression is not a causal risk factor for future disease progression, but a reflection of prior disease activity and present severity. Although these data do not support a causal role for depression in influencing subsequent disability outcomes, the high prevalence of depressive illness in this cohort underscores the importance of recognising and treating depression in people with MS^18^.

## Data Availability

All code used in these analyses is available at https://github.com/benjacobs123456/UKMSR_depression. Access to UKMSR data is open to all researchers on application and subject to suitable governance review. Details of how to apply for the data can be found here: https://ukmsregister.org/Research/WorkingWithUs.
